# Older people’s goals of care in relation to frailty status—the COOP-study

**DOI:** 10.1093/ageing/afae097

**Published:** 2024-05-25

**Authors:** Veerle M G T H van der Klei, Yvonne M Drewes, Bas F M van Raaij, Maaike D W van Dalsen, Anneke G Julien, Jan Festen, Harmke Polinder-Bos, Simon P Mooijaart, Jacobijn Gussekloo, Frederiek van den Bos, the COVID-19 Outcomes in Older People (COOP)-consortium, Karel G M Moons, Maarten van Smeden, Kim Luijken, Petra Elders

**Affiliations:** Department of Internal Medicine, Section Gerontology and Geriatrics, Leiden University Medical Center, Leiden, the Netherlands; LUMC Center for Medicine for Older People (LCO), Leiden University Medical Center, Leiden, the Netherlands; Department of Internal Medicine, Section Gerontology and Geriatrics, Leiden University Medical Center, Leiden, the Netherlands; LUMC Center for Medicine for Older People (LCO), Leiden University Medical Center, Leiden, the Netherlands; Department of Public Health and Primary Care, Leiden University Medical Center, Leiden, the Netherlands; Department of Internal Medicine, Section Gerontology and Geriatrics, Leiden University Medical Center, Leiden, the Netherlands; LUMC Center for Medicine for Older People (LCO), Leiden University Medical Center, Leiden, the Netherlands; Department of Internal Medicine, Section Gerontology and Geriatrics, Leiden University Medical Center, Leiden, the Netherlands; LUMC Center for Medicine for Older People (LCO), Leiden University Medical Center, Leiden, the Netherlands; Department of Internal Medicine, Section Gerontology and Geriatrics, Leiden University Medical Center, Leiden, the Netherlands; LUMC Center for Medicine for Older People (LCO), Leiden University Medical Center, Leiden, the Netherlands; KBO-PCOB, Nieuwegein, the Netherlands; Department of Geriatrics, Erasmus Medical Center, Rotterdam, the Netherlands; Department of Internal Medicine, Section Gerontology and Geriatrics, Leiden University Medical Center, Leiden, the Netherlands; LUMC Center for Medicine for Older People (LCO), Leiden University Medical Center, Leiden, the Netherlands; Department of Internal Medicine, Section Gerontology and Geriatrics, Leiden University Medical Center, Leiden, the Netherlands; LUMC Center for Medicine for Older People (LCO), Leiden University Medical Center, Leiden, the Netherlands; Department of Public Health and Primary Care, Leiden University Medical Center, Leiden, the Netherlands; Department of Internal Medicine, Section Gerontology and Geriatrics, Leiden University Medical Center, Leiden, the Netherlands; LUMC Center for Medicine for Older People (LCO), Leiden University Medical Center, Leiden, the Netherlands

**Keywords:** older people, decision-making, patient preference, health outcome prioritisation, patient and public involvement

## Abstract

**Background:**

Literature relating older people’s goals of care to their varying frailty status is scarce.

**Objective:**

To investigate goals of care in case of acute and/or severe disease in relationship to frailty status among the general older population.

**Method:**

Older people aged ≥70 in the Netherlands completed a questionnaire. They were divided into three subgroups based on a self-reported Clinical Frailty Scale: fit (CFS 1–3), mildly frail (CFS 4–5) and severely frail (CFS 6–8). Seven goals were graded as unimportant (1–5), somewhat important (6–7) or very important (8–10): extending life, preserving quality of life (QoL), staying independent, relieving symptoms, supporting others, preventing hospital admission and preventing nursing home admission.

**Results:**

Of the 1,278 participants (median age 76 years, 63% female), 57% was fit, 32% mildly frail and 12% severely frail. Overall, participants most frequently considered preventing nursing home admission as very important (87%), followed by staying independent (84%) and preserving QoL (83%), and least frequently considered extending life as very important (31%). All frailty subgroups reported similar preferences out of the surveyed goals as the overall study population. However, participants with a higher frailty status attached slightly less importance to each individual goal compared with fit participants (*P*_trend_-values ≤ 0.037).

**Conclusion:**

Preferred goals of care are not related to frailty status, while the importance ascribed to individual goals is slightly lower with higher frailty status. Future research should prioritise outcomes related to the shared goals of fit, mildly frail and severely frail older people to improve personalised medicine for older patients.

## Key Points

- Patients’ goals of care are essential to be addressed in shared decision-making, especially with older people.
- Frailty in older people is associated with adverse outcomes, but its relationship to preferred outcomes or goals is hardly known.
- Fit, mildly frail and severely frail older people (70+) surprisingly preferred similar goals of care when being ill.
- This finding extends previous literature on preferred goals of care to the general older population.
- Research should prioritise outcomes related to these shared goals to improve personalised medicine, independent of frailty.

## Introduction

Population ageing causes a worldwide increase in the number of older people [1], with a simultaneous increase in multi-morbidity [2]. Health care professionals across the health care system are therefore challenged with complex care decisions [3]. Evidence-based medicine for older patients is complex due to the lack of relevant scientific evidence to substantiate treatment recommendations [4]. For instance, only 8% of clinical trials are specifically designed for older people [4, 5]. Furthermore, there is a high degree of heterogeneity in older people’s health status (functional, somatic, mental and social) [6] and frailty status to be considered. Frailty is their vulnerability to health status changes triggered by relatively minor stressors [3, 7]. Subsequent uncertainty about the best practices in treating older patients stresses the importance of shared decision-making (SDM) to provide personalised medicine in old age [4], namely to elicit older patients’ goals of care and to align health care accordingly [8–10].

Goals of care vary widely among older people. Many older patients prioritise independence, while some prefer life extension [11–13]. Others primarily value goals such as quality of life (QoL), being comfortable or staying out of the hospital [13–15]. Unfortunately, numerous barriers hamper the elicitation and incorporation of goals of care in medical practice (e.g. limited time for SDM or lack of continuity of care) [16–18], as recently emphasised during the COVID-19 pandemic [19]. Goals of care may also vary in importance over time, for instance, influenced by family [20]. Health care professionals are therefore prone to be mistaken about older patients’ goals of care [9, 13] and older patients are prone to receive health care which does not align with their true preferred goals [4, 16].

Frailty status may explain some variety in the goals of care of older people, as prioritisation of goals seems, for instance, driven by disease experiences [13, 20–22]. While frailty in older people is known to be associated with adverse outcomes (e.g. decline in functioning, nursing home admission and mortality) [7], literature relating varying frailty status to older people’s preferred outcomes or goals of care is scarce. To the best of our knowledge, only two studies have explored this relationship as yet and both found no association between frailty status and older people’s single most important goal of care [11, 12]. However, these studies included a specific subset of older people (patients with severe disease, mostly cancer) and considered few goals of care (e.g. omitting QoL). We therefore investigated the goals of care in case of acute and/or severe disease in relationship to frailty status among the general older population. We hypothesised that the preferred goals of care of older people would differ with increasing frailty status, including decreased importance of life extension. As frailty assessment has a central role in complex care decisions in old age [3], knowing this relationship may reduce differences in perspectives on their true preferred goals and improve subsequent care alignment.

## Methods

### Study design and participants

This study is a cross-sectional, quantitative study as part of a mixed-methods study on older people’s goals of care, which is embedded in the *COVID-19 Outcomes in Older People* (COOP)-consortium in the Netherlands. Anyone aged 70 years or older and living in the Netherlands could participate. We aimed to include at least 100 participants per frailty subgroup for group comparisons [14, 23]. Data were collected from May up to October 2022. The anonymous questionnaire was distributed online by senior organisations and onward distribution was encouraged (i.e. the snowball method). In addition, a hard copy of the questionnaire was actively distributed by several health care and welfare professionals (e.g. at in- and outpatient clinics, primary care practices, community centres, libraries and nursing homes). If required or desired, someone was allowed to assist in filling out the questionnaire. Most questions were mandatory, minimising missing data, and only unique data entries with sufficient questions completed were included in the analysis. The study was approved by the Institutional Review Board of the Leiden University Medical Center for observational COVID-19 studies (2022–005). See Appendix S1A and B for a more detailed account of our methodology.

### Questionnaire

#### Exposure: frailty status

Frailty was assessed by 11 self-reported, closed-ended questions aimed at deriving the Clinical Frailty Scale (CFS) [24], as the CFS was widely implemented throughout the COVID-19 pandemic [25, 26]. Participants were divided into three subgroups: fit (CFS 1–3), mildly frail (CFS 4–5) and severely frail (CFS 6–8), and were excluded if terminally ill (CFS 9), as they were outside the scope of our research question. See Appendix S1C for more details on our self-reported CFS approach.

#### Outcome: goals of care

Goals of care were defined as the overarching aims of medical care for a patient [27]. Participants were surveyed about their goals of care in case of hypothetical acute and/or severe disease, henceforth referred to as goals. See Appendix S1D for the questionnaire and its substantiation [14, 28]. The goals extending life, preserving QoL, staying independent, relieving symptoms, supporting others, preventing hospital admission and preventing nursing home admission were assessed in three ways.

Firstly, participants graded each of these goals individually on a Likert scale of 1 (not important at all) to 10 (extremely important), which was subsequently categorised as unimportant (1–5), somewhat important (6–7) and very important (8–10). This grading was evaluated ‘across all goals’ to explore the preferences of the overall study population out of these seven goals. In the same way, the preferred goals of the fit, mildly frail and severely frail subgroups were evaluated. Secondly, the trend in grading across the three frailty subgroups was evaluated ‘for each goal separately’ to explore the amount of importance attached to each individual goal. Thirdly, participants were asked to compare the goals against each other by indicating which single goal was most or least important. Lastly, participants could phrase one additional goal themselves as an answer to an open question. The content of these answers was thematically categorised in a consensus meeting by two researchers (VvdK and FvdB).

#### Other measures

See Appendix S1E for the definition of other measures, including experienced health problems [6].

### Patient and public involvement

Our Seniors Advisory Board (10 community-dwelling older people) was involved throughout the entire research cycle according to varying roles of the Involvement-Matrix: listener, co-thinker, advisor, partner and decision-maker [29]. Their involvement especially improved the study’s inclusiveness for the heterogenous older population. Furthermore, a sensitivity analysis was added replacing the self-reported CFS by mental and social health problems, because they hypothesised that the CFS primarily reflected somatic health instead of psychosocial well-being. See Appendix S2 for a detailed account of patient and public involvement [30].

### Statistical analysis

Non-normally distributed continuous data were presented as medians with interquartile ranges (IQR) and categorical data were presented as proportions. Results were stratified by frailty status and trends across groups were tested by Jonckheere-Terpstra and linear-by-linear Chi-square tests, respectively. Those with and without mental and social health problems were compared by use of Pearson Chi-square tests. SPSS Statistics version 25.0 was used (IBM Corp, Armonk, NY) and a *P*-value <0.05 was considered statistically significant.

## Results

### Participants’ characteristics

Out of the 1,294 unique questionnaires with sufficient questions completed, 16 questionnaires were excluded because participants had a self-reported CFS score 9 (terminally ill). The 1,278 included participants were 70–103 years of age (median 76 years old, IQR 73–80) (Table 1). The majority was female (63%) and nearly half the study population had a lower or middle-level education (47%). Altogether, 45% lived alone, 7.5% used home or informal care and 5.2% resided in an assisted living facility or nursing home. The experience of health problems ranged from 9.1% in the functional domain to 39% in the mental domain and 28% had a previous COVID-19 infection. With respect to frailty status, 725 participants were classified as fit (57%), 404 as mildly frail (32%) and 149 as severely frail (12%).

**Table 1:** Characteristics of study participants, overall and stratified by frailty status

	Overall *N* = 1,278	Fit *N* = 725	Mildly frail *N* = 404	Severely frail *N* = 149	*P*-value
Age, median (IQR), years	76 (73, 80)	75 (72, 78)	77 (73, 82)	80 (75, 86)	<0.001
Female, *n* (%)	797 (63)	426 (59)	275 (68)	96 (64)	0.016
Migration background, *n* (%)^*^	100 (7.8)^a^	54 (7.4)	30 (7.4)	16 (11)	0.288
Lower or middle educated, *n* (%)^†^	602 (47)	307 (43)	192 (48)	103 (69)	<0.001
Experience with current income, *n* (%)					
Comfortable	684 (56)	440 (63)	184 (48)	60 (44)	<0.001
Reasonable	477 (39)	234 (34)	176 (46)	67 (49)	
Difficulties	56 (4.6)	22 (3.2)	23 (6.0)	11 (8.0)	
Religion considered important, *n* (%)	399 (33)	201 (29)	135 (37)	63 (44)	<0.001
Living alone, *n* (%)	579 (45)	278 (38)	214 (53)	87 (58)	<0.001
Assisted living or nursing home, *n* (%)	66 (5.2)	11 (1.5)	22 (5.4)	33 (22)	<0.001
Use of home or informal care, *n* (%)	96 (7.5)	2 (0)	15 (3.7)	79 (54)	<0.001
Previous COVID-19 infection, n (%)	361 (28)	205 (28)	113 (28)	43 (29)	0.920
Health problems, *n* (%)^‡^					
Somatic domain	473 (37)	129 (18)	222 (55)	122 (83)	<0.001
Mental domain	497 (39)	195 (27)	209 (52)	93 (63)	<0.001
Social domain	402 (32)	161 (22)	169 (42)	72 (49)	<0.001
Functional domain	116 (9.1)	0 (0)	21 (5.2)	95 (65)	n/a
Any assistance in filling out this questionnaire, *n* (%)	96 (7.9)	11 (1.6)	32 (8.3)	53 (38)	<0.001

^*^Migration background was defined as country of birth outside the Netherlands. Out of those with a migration background in our study population, the country of birth was: 27% Indonesia, 25% Surinam, 20% another European country, 7% Dutch Antilles, 5% Turkey, 4% Morocco and 12% originated from other countries.

^†^Educational attainment was defined as a lower or middle highest completed level of education (i.e. none, primary or secondary (vocational)) compared with a higher completed level of education (i.e. higher vocational or university) according to the Dutch Verhage scale [31].

^‡^Health problems were defined as experiencing two or more deficits during the past month out of the four to seven deficits questioned per health domain of the Integrated Systematic Care for Older People (ISCOPE)-screening questionnaire [6].

**Notes**: missing data (*n*): sex (3), living alone (1), use of care (3), education (5), experience with current income (61), religion (85), corona-infection (13). Somatic domain (7), mental domain (5), social domain (2), functional domain (3).

With a higher frailty status (Table 1), participants were older, were more often female and had a lower level of completed education (*P*_trend_-values ≤ 0.016). With a higher frailty status, participants were also more likely to live alone, use care and/or reside in assistant living or nursing homes (*P*_trend_-values < 0.001). Compared with the other two frailty subgroups, severely frail participants experienced health problems more frequently in the somatic domain (83% vs. 55% of mildly frail and 18% of fit participants), in the mental domain (63% vs. 52% of mildly frail and 27% of fit participants) and in the social domain (49% vs. 42% of mildly frail and 22% of fit participants) (all *P*_trend_-values ≤0.001).

### Goals of care

**Figure 1:**
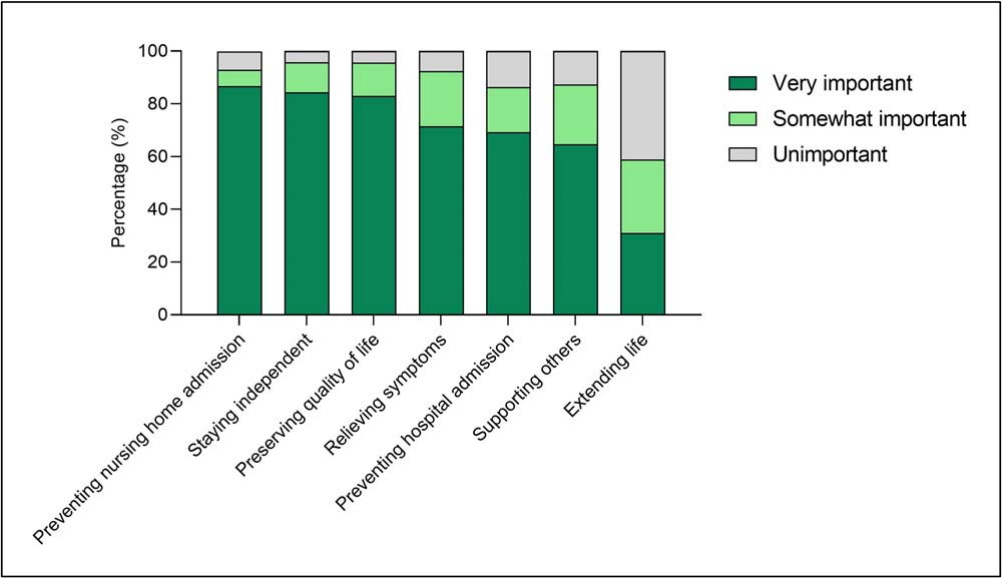
Goals of care of the overall study population (*n* = 1,278). **Notes:** missing data (*n*): extending my life (11), preserving my QoL (6), staying as independent as possible (5), relieving my symptoms (13), remaining to support others (12), preventing hospital admission (12) and preventing nursing home admission (29, of which 21 were already living in a nursing home, which means this goal was not applicable to them anymore).

**Figure 2:**
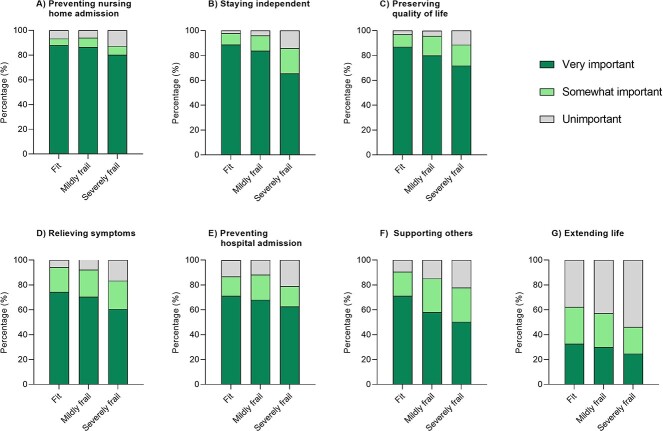
Goals of care of older people stratified by frailty status: fit (*n* = 725), mildly frail (*n* = 404) and severely frail (*n* = 149). **Notes**: Trends across frailty subgroups in the importance attached to each goal were tested by linear-by-linear Chi Square tests. All p-trend values were ≤0.037.

Firstly, in the exploration of preferences out of the seven goals that were individually graded, the overall study population most frequently considered preventing nursing home admission as very important (87%), closely followed by staying independent (84%) and preserving QoL (83%). In contrast, the overall study population least frequently considered extending life as very important (31%) (Figure 1). These preferred goals were independent of frailty status, as the fit, mildly frail and severely frail participants considered the same goals as most and least frequently very important as the overall study population (Figure 2).

Secondly, in the evaluation of the importance attached to each goal, participants with a higher frailty status on average considered all seven goals to be of slightly less importance compared with fit participants (Figure 2; all *P*_trend_-values ≤ 0.037). For instance, severely frail participants less frequently considered staying independent (66% vs. 84% of mildly frail and 89% of fit participants) and supporting others (50% vs. 58% of mildly frail and 71% of fit participants) as very important, and more frequently considered extending life as unimportant (54% vs. 43% of mildly frail and 38% of fit participants).

Thirdly, when asked to compare the goals (as opposed to grading them individually as in the preceding paragraphs), the overall study population most frequently considered preserving QoL as the single most important goal (51%). This preference was independent of frailty status, as all frailty subgroups most frequently considered preserving QoL as the single most important goal (41% of the severely frail, 47% of the mildly frail and 56% of the fit participants; Appendix S3).

Lastly, 97 participants (7.6%) phrased a goal as an answer to our open question. Categorising these answers showed that most answers were directly related to our surveyed goals (*n* = 69, 71%). Answers related to a dignified end of life and dying (*n* = 25, 26%) were the most evident addition to our surveyed goals in the overall study population, as well as in all frailty subgroups (not shown).

#### Sensitivity analyses

Sensitivity analyses stratifying by mental and social health problems instead of frailty status showed similar results on goals of care (Appendix S4). Only the trends in the importance attached to each goal were less consistent across the seven goals. For instance, those with and without social health problems equally rated staying independent as very important (85% vs. 84%, respectively).

## Discussion

### Principal findings

In this explorative, quantitative study among the general older population, we showed that preferred goals of care in case of acute and/or severe disease were not related to frailty status. Fit, mildly frail and severely frail older people predominantly considered preventing nursing home admission, staying independent and preserving QoL as very important goals, as well as most frequently considered preserving QoL as their single most important goal out of the surveyed goals. Similarly, all frailty subgroups considered extending life as relatively unimportant. The importance attached to each goal separately was, however, related to frailty status, as older people with a higher frailty status on average attached slightly less importance to each individual goal than fit older people.

### Comparison with literature

Previous studies on goals of care among the older population focused on specific subsets of older people: those living with frailty [14] or multimorbidity [13, 32, 33] or patients facing severe disease [11, 12, 15, 33]. We primarily add to this body of literature by including the general older population and by comparing large subgroups along the wide spectrum of frailty ranging from fit to severely frail.

When investigating the relationship between goals of care and frailty status, our finding that fit, mildly frail and severely frail older people prefer similar goals of care is rather surprising, as disease experience and care needs accumulate with higher frailty status [7]. A recent systematic review that focused on older people’s preferences for different long-term care options also showed that poor health status was positively associated with the willingness to use institutional care services [22]. However, our results are consistent with the two previously mentioned Dutch studies (*n* = 170 and *n* = 350) that specifically focused on goals of care in relationship to frailty and that found no association in older patients with severe disease (mostly cancer) which were referred to the geriatric outpatient clinic [11, 12]. We extend their findings to a hypothetical situation in the general older population.

When diving into which goals of care matter to older people when being ill, we replicate findings on the paramount importance of ageing in place [22, 32], independence [11–14, 32, 33] and QoL [14, 33], as well as the relative unimportance of life extension [14, 15, 32], and extend them to the general older population. In contrast, the utmost prioritisation of preserving QoL seems more unified in our community-based sample (51%) than in frail older people following recent acute illness (15%), who most frequently considered preventing hospital admission as their single most important goal (20%) [14]. A dignified end-of-life and dying, as the additional goal of care phrased by older people themselves in our study, was either not measured [11–15] or did not emerge [32, 33] in other studies.

The importance attached to individual goals has not previously been studied in relationship to frailty status, and there is no known cut-off which defines a clinically relevant difference between subgroups.

### Possible explanations

On the one hand, the similarities in preferred goals of care between fit, mildly frail and severely frail older people could reflect the stability of cross-disease goals (e.g. ageing in place) [34] and their underlying values (e.g. autonomy) [14], as opposed to the susceptibility to change of disease-specific goals (e.g. reaching a target blood pressure level) [34]. Therefore, our findings could reflect shared goals of the general older population. On the other hand, the similarities in preferred goals of care between frailty subgroups in this quantitative study do not necessarily imply that there are no differences in preferences based on frailty status at all. Fit, mildly frail and severely frail older people might ascribe different meaning to same goals (e.g. what one needs in order to feel independent) [21], which we are currently exploring in a subsequent interview study. Alternatively, variation in older patients’ goals of care might better be explained by their outlook on life instead of actual frailty or health status [12], which would be worthwhile to explore in the general older population.

Whether older people with a higher frailty status indeed attach a slightly lower importance to all goals than fit older people should be interpreted with caution. It could be explained by higher acceptance of a poor prognosis among frail older people (e.g. modesty in grading goals) [35]. Frail older people might also grade hypothetical situations differently than their fit counterparts based on more real-life experience with competing goals when being ill (e.g. grading less goals as very important) [34]. Wishes to avoid being a concern or burden to family, which are known to influence preferences [20], might have a more prominent role in case of frailty and related functional dependency than in fit older people, especially if one was assisted in filling out the questionnaire (38% of severely frail vs. 8.3% of mildly frail and 1.6% of fit participants). In case of cognitive complaints, older people may also experience difficulty with hypothetical thinking, as well as forming and expressing preferred goals [20].

### Implications

In any case, the outcomes examined in medical research mostly do not correspond to the goals of care that matter most to the vast majority of older patients across our health care systems, as studies predominantly focus on disease- or treatment-related outcomes instead of patient-related outcomes. For example, while 81% of trials about common types of cancer (2005–2020) focused on progression-free survival, merely 21% addressed QoL, functioning and/or health care utilisation and even less than 1% focused on one of these outcomes as the primary objective [36]. To provide person-centred and value-based health care, however, what matters most to patients should be at the heart of medical research, practice and policy. The importance of QoL, ageing in place and independence to the general older population in our study could serve as a new standard for outcomes in medical research on acute and/or severe disease instead of the conventional focus on survival. Moreover, these results provide health care professionals with relevant knowledge on the likely goals of care of older patients, as a much-needed, data-based starting point for advanced care planning [37], as well as shared-decision making with individual older patients [9], independent of their frailty status.

### Strengths and limitations

To our knowledge, we are the first to specifically study the relationship between older people’s goals of care and their varying frailty status in a broad community sample, and we addressed this research priority rapidly in response to the COVID-19 pandemic. Our use of an anonymous, accessible questionnaire and close collaboration with our Seniors Advisory Board enabled the inclusion of a wide variety of older people across the Netherlands, especially regarding frailty, which broadens the potential generalizability of our findings. We addressed frailty among this older population by use of a self-reported, pilot-tested version of the CFS, which holds great potential for use by older people in medical practice. Furthermore, we gave a voice to older people to show their perspective on goals of care as an essential contribution to the prevention of inappropriate care in old age.

This study also has several limitations. In line with previous literature [13–15, 32], our results on older people’s goals of care regard hypothetical decision-making, which may change in case of actual disease [13, 14] and related emotions [8]. It also regards a cross-sectional study and therefore cannot account for changes in goals of care over time [20]. Both are not barriers to the intended aim of our study but do emphasise the ongoing need for SDM. Secondly, this quantitative study did not account for potential interrelationships between the goals, which are, however, investigated in our subsequent interview study. Thirdly, the self-reported CFS should be studied further for construct validity (e.g. against the professional-reported CFS) and test–retest reliability. Studies in the emergency department previously showed moderate differences between CFS-scores reported by older patients and professionals [38, 39]. For instance, older patients more often regarded themselves as ‘very fit’ (CFS = 1). To what extent such disagreement applies to our study remains unknown. Instead of circling a pictograph and clinical description of frailty in an acute, care-dependent setting [38, 39], we used a self-reported approach largely based on validated self-reported questions for a community-based study population [6]. As our subsequent frailty prevalence was similar to self-reported frailty in a large population-based study in the Netherlands [40] and our frailty subgroups showed expected trends in socio-demographics, as well as health problems [41], we consider it unlikely that the self-reported CFS affected our results.

## Conclusion

Preferred goals of care are not related to frailty status among the general older population, while the importance ascribed to individual goals is slightly lower with higher frailty status. Fit, mildly frail and severely frail older people prefer preventing nursing home admission, staying independent and preserving QoL as most important goals when being ill, while life extension is relatively unimportant. Future research should prioritise outcomes related to these shared goals of care to improve personalised medicine for older patients, independent of their frailty status.

## Supplementary Material

aa-23-2162-File004_afae097

## Data Availability

The data are not publicly available due to privacy concerns. The data that support the findings of this study are available upon reasonable request from the corresponding author.
