# Supplementary material for: Older people’s goals of care in relation to frailty status—the COOP-study
Source: Age Ageing. 2024 May 25;53(5):afae097. doi: 10.1093/ageing/afae097 (PMC11127771; doi:10.1093/ageing/afae097)
Supplement: aa-23-2162-File004_afae097 [file aa-23-2162-file004_afae097.docx]

**Appendices: Older people’s goals of care in relationship to frailty status – the COOP study.**

**Page of contents:**

**Appendix 1**. Additional information on the methods:

**1A.** Context of the COOP-study on goals of care

**1B.** Data collection and processing

**1C.** Our self-reported CFS approach

**1D.** Substantiation of goals of care

**1E.** Definition of other measures

**Appendix 2.** Patient and Public Involvement (PPI) in the COOP quantitative substudy on older

people’s goals of care according to the GRIPP2 short form

**Appendix 3.** Prioritization of the most and least important goals of care,

overall and stratified by frailty status

**Appendix 4.** Sensitivity analysis: goals of care stratified by mental and social health problems

**References**

**Appendix 1A. Context of the COOP-study on goals of care**

This study is a cross-sectional, quantitative study as part of a mixed-methods study on older people’s goals of care in case of acute and/or severe disease, initiated in response to the COVID-19 pandemic. It is embedded in the *COVID-19 Outcomes in Older People* (COOP)-consortium in the Netherlands: a national collaboration between researchers and health care professionals from different care settings (hospitals, primary care practices, and nursing homes), and a Seniors Advisory Board. Our study used this collaborative infrastructure, but is otherwise unrelated to its COVID-19-specific study objectives [1].

At the time of our data collection (May-October 2022), practically all preventive measures of the COVID-19 pandemic were abolished in the Netherlands (e.g. no more lockdowns, social distancing or obligatory use of face masks) [2] and approximately 90% of people aged 70 years and over were repeatedly vaccinated against COVID-19 [3]. Throughout the preceding waves of COVID-19, vivid illustrations of being acutely and/or severely ill had dominated the media [4] and advanced care planning gained more relevance amongst both older people [5, 6], as well as health care professionals [7].

**Appendix 1B. Data collection and processing**

The anonymous questionnaire was created with Qualtrics software [8] and distributed online in a similar manner as our previous study during the COVID-19 pandemic [9, 10]: older people were invited through emails, newsletters, magazines and/or social media of national, regional and local senior organizations. In addition, a hard copy version was actively distributed by several health care and welfare professionals to include sufficient frail older people. We additionally targeted older people with a low socio-economic status and older immigrants to improve diversity of the study population.

Filling out the questionnaire following introductory information was considered informed consent. As most questions of the online version were mandatory to fill out, missing data were minimized. Only participants who completed more than half of the questionnaire were included in the analysis to ensure any data availability on the exposure and the outcome. In other words, to assign a frailty subgroup and to evaluate any grading on goals of care. Simultaneous availability of descriptives such as postal code also minimized probable duplicate data entries. These duplicates may have originated from multiple (incomplete) attempts by the same individual in case of online technical difficulties and were removed after duplicate screening in a consensus meeting by two researchers (VvdK and FvdB).

**Appendix 1C. Our self-reported CFS approach**

In the absence of a validated, self-reported Clinical Frailty Scale (CFS)-research instrument [11], we used the functional domain of the validated Integrated Systematic Care for Older People (ISCOPE)-screening questionnaire (five questions to assess functional deficits in the general older population) [12], complemented by extra response options and questions on functioning, physical activity, symptoms, and prognosis. These additions were based upon two CFS-tools for inexperienced raters [13, 14], which we regarded older people to be as well, and on consensus with the Seniors Advisory Board and geriatricians. In line with ISCOPE [12], participants were surveyed about their situation during the past month (i.e. extending the regular CFS-period of two weeks prior to development of acute disease) [15]. See **Table Appendix 1C** for the applied questions for the self-reported CFS.

In line with the above-mentioned CFS-tools [13, 14], there was a hierarchy in the assignment of participants to frailty subgroups in our self-reported approach: from severely frail (CFS 6-8) to mildly frail (CFS 4-5) and fit (CFS 1-3). Participants were assigned to one of these frailty subgroups when meeting one or more CFS-descriptions of that subgroup. See **Figure Appendix 1C** for details on the applied algorithm to derive the self-reported CFS from the questions in **Table Appendix 1C**.

| **Table Appendix 1C**. Questionnaire aimed at deriving the self-reported Clinical Frailty Scale. | | | |
| --- | --- | --- | --- |
| **All questions regard your daily life of the past month. The first seven questions relate to how you function/manage day to day life. “Independently” means without the help of anyone else. You may however be helped in these activities by aids such as a stick, walking frame, or wheelchair.** | | | |
| 1. Can you dress and undress yourself independently?*  - Yes - No, I require minimal assistance (e.g. assistance with my support stockings, occasional cuing on what to do or having someone else on standby) - No, I require more or full assistance. | | | |
| 1. Can you go to the toilet independently?^†^ | | | |
| - Yes | | - No | |
| 1. Can you shower independently?^‡^  - Yes - No, I require minimal assistance (e.g. assistance with washing my hair, scrubbing my back and/or drying my toes) - No, I require more or full assistance. | | | |
| 1. Can you run errands independently?^†^ | | | |
| - Yes | | - No | |
| 1. Can you walk outdoors independently?^†^ | | | |
| - Yes | | - No | |
| 1. Can you manage your finances independently (collect your money, pay your bills)?^†^ | | | |
| - Yes | | - No | |
| 1. Can you do your housekeeping independently?^‡^  - Yes - No, I require minimal assistance (e.g. with heavy housework such as vacuuming). - No, I require more or full assistance. - Never done, always by others. | | | |
| 1. Which description of physical activity (e.g. exercising, hiking or riding a bike) suits you best?^‡^  - I am regularly physically active (e.g. weekly or daily). - I am occasionally physically active (e.g. seasonally). - I am not physically active beyond daily life activities. | | | |
| 1. Do you experience symptoms which limit your physical activity (e.g. being slowed up or being tired which complicates running errands)?^‡^ | | | |
| - Yes | - Sometimes | - No |  |
| 1. Are there activities in daily life which are not going well, because you (sometimes) have memory complaints?^‡^ | | | |
| - Yes | - Sometimes | - No |  |
| 1. Has your doctor ever told you, you probably do not have a long time to live anymore?^‡^ | | | |
| - Yes | - No | - Rather not answer |  |
| **Notes**: ^*^Original question of the ISCOPE-questionnaire complemented by extra response options. ^†^Original question of the ISCOPE-questionnaire. ^‡^Additional questions specifically added to derive the self-reported CFS-score.  **Abbreviations**: CFS, Clinical Frailty Scale; ISCOPE, Integrated Systematic Care for Older People; COOP, COVID-19 Outcomes in Older People. | | | |

| 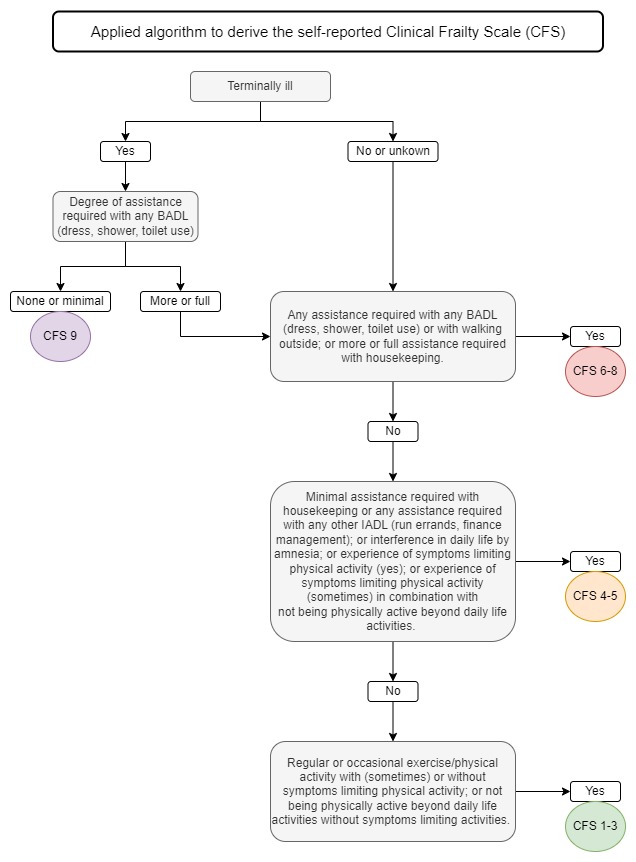 |
| --- |
| **Figure Appendix 1C.** Applied algorithm to derive the self-reported Clinical Frailty Scale. |
|  |
| **Notes:** there was a hierarchy in the self-reported CFS-assignment (from severely frail (CFS 6-8) to mildly frail (CFS 4-5) and fit (CFS 1-3)), and participants were assigned to one of these frailty subgroups when meeting one or more CFS-descriptions of that subgroup. The text in this figure corresponds verbatim to the answer options of the eleven questions aimed at deriving the self-reported CFS in Table Appendix 1C. **Abbreviations:** BADL, Basic Activities of Daily Living; IADL, Instrumental Activities of Daily Living; CFS, Clinical Frailty Scale. |

**1D. Substantiation of goals of care**

Seven goals of care were assessed. Based on a previous study in frail older people [16] and its theoretical framework [17], we included the following goals: extending life, preserving QoL, staying independent, relieving symptoms, supporting others, and preventing hospital admission. Based on consensus with geriatricians and the Seniors Advisory Board, we added the goal preventing nursing home admission to adapt the questionnaire to the Dutch context. See **Table Appendix 1D** for the questionnaire. The cut-offs applied to the grading were chosen based on consensus among a diverse group of experts of the COOP-consortium; there was no specific substantiation in the literature [16].

| **Table Appendix 1D.** Questionnaire on goals of care. |
| --- |
| **The following 3 questions regard your goals of care in medical decisions in case of acute and/or severe disease.** |
| 1. In situations of acute and/or severe disease, treatment decisions may need to be made. In this situation, how important would each of the following goals of care be to you?   ***( 1 not important at all – 10 extremely important )***   1. Extending my life.   € 1 € 2 € 3 € 4 € 5 € 6 € 7 € 8 € 9 € 10   1. Preserving my quality of life.   € 1 € 2 € 3 € 4 € 5 € 6 € 7 € 8 € 9 € 10   1. Staying as independent as possible.   € 1 € 2 € 3 € 4 € 5 € 6 € 7 € 8 € 9 € 10   1. Relieving my symptoms.   € 1 € 2 € 3 € 4 € 5 € 6 € 7 € 8 € 9 € 10   1. Supporting others close to me.   € 1 € 2 € 3 € 4 € 5 € 6 € 7 € 8 € 9 € 10   1. To stay out of a hospital.   € 1 € 2 € 3 € 4 € 5 € 6 € 7 € 8 € 9 € 10   1. To stay out of a nursing home.   € 1 € 2 € 3 € 4 € 5 € 6 € 7 € 8 € 9 € 10   1. Other, namely ……………………………………………………………………………………………………   € 1 € 2 € 3 € 4 € 5 € 6 € 7 € 8 € 9 € 10 |
| 1. Which of these goals is the most important to you? ***( Report 1 letter: A – H )***   ……………………………………………………………………………………………………………………………….. |
| 1. Which of these goals is the least important to you? ***( Report 1 letter: A – H )***   ……………………………………………………………………………………………………………………………….. |
|  |

**Appendix 1E. Definition of other measures**

Socio-demographic data was gathered on: age, sex, migration background (country of birth outside the Netherlands), educational attainment (a lower or middle vs. a higher completed level of education according to the Dutch Verhage scale) [18], experience of current income (comfortable vs. reasonable vs. difficulties), importance of religion (important vs. not important), living situation (alone vs. with others; independent vs. assisted living or nursing home), and use of home or informal care. Participants reported previous COVID-19 infections, as well as deficits across the other domains of the Integrated Systematic Care for Older People (ISCOPE)-screening questionnaire: somatic, mental, and social health besides functional health [12]. Health problems were defined as experiencing two or more deficits during the past month out of the four to seven deficits questioned per domain.

| **Appendix 2.** Patient and Public Involvement (PPI) in the COOP quantitative substudy on older people’s goals of care according to the GRIPP2 short form [19]. | |
| --- | --- |
| **1. Aim**  Report the aim of PPI in the study | To give voice to older people who are ‘experts by experience’ based on their age, diverse medical histories and experience with the COVID-19 pandemic in the Dutch context by collaborating with them in all research stages of this exploration of goals of care in relationship to frailty status. |
| **2. Methods**  Provide a clear description of the methods used for PPI in the study | Ten older people were recruited to participate in the COOP Seniors Advisory Board: median age 75 years old (IQR 70-78), 70% female and 100% higher educated*. They had diverse medical (e.g. history of COVID-19 or other disease and ranging from fit to mildly frail) and professional backgrounds (health care and research, anthropology, education and finances). 70% also represented another senior organization spread across the Netherlands.  Throughout the project the Board participated according to all five roles of the Involvement-Matrix: listener, co-thinker, advisor, partner and decision-maker [20]. Their chairman was part of the steering committee of the larger COOP-consortium and was co-leader of this substudy on goals of care. He was involved from the very beginning in defining the research question and drafting the grant application. Subsequently, the Board was involved in the study design, in carrying out pilots from the lay perspective, in data collection and in interpretation of the results. They will also be involved in dissemination of a lay summary of our findings via senior organizations once the currently ongoing, qualitative substudy is finished.  The Board had bi-monthly meetings either online or in person with at least one junior and one senior researcher from this substudy involved. |
| **3. Results**  Outcomes—Report the results of PPI in the study, including both  positive and negative outcomes | The COOP Seniors Advisory Board influenced this substudy as follows:   - The Board hypothesized that the Clinical Frailty Scale (CFS) primarily reflected somatic health instead of a more holistic concept of frailty, while they regard psychosocial well-being as very important when indicating heterogeneity in old age as well. To complement our analyses with the primary exposure (frailty according to the self-reported CFS), the exposure was replaced by the measures mental and social health problems in a sensitivity analysis.      - The outcome measure was adapted to the Dutch context by addition of the goal ‘preventing nursing home admission’. - Older people with a lower completed level of education and/or older immigrants participated in the pilot of the questionnaire. - The language and lay-out used in recruitment of participants and in the questionnaire were revised to improve comprehension, readability and, for the online questionnaire, user friendliness. - The Board distributed the questionnaire in their personal networks and via senior organizations (if applicable), as well as proposed strategies to reach a heterogenous older study population. - Their interpretation of results was incorporated in the discussion based on Board meetings and co-authorship of the chairman. - Joint representation of the Board and researchers was present at multiple national conferences on geriatric care in the Netherlands.   For pragmatic reasons, some suggestions of the Board could unfortunately not be implemented. For example, the Board advised to incorporate more nuances in the questions on experienced health problems, which were not adjusted as those questions originated from a validated questionnaire [12]. |
| **4. Discussion and**  **Conclusions**  Outcomes—Comment on the extent to which PPI influenced the study  overall. Describe positive and negative effects | Involvement of older people in this substudy encompassed varying roles of the Involvement-Matrix throughout all research stages and especially enhanced the study’s inclusiveness for the heterogenous older population. Due to the quantitative nature of this substudy not all suggestions of the Board could be fully incorporated, but the subsequent qualitative substudy allows further involvement with more nuances and personal perspectives. |
| **5. Reflections,**  **critical perspective**  Comment critically on the study, reflecting on the things that went well  and those that did not, so others can learn from this experience | The involvement of the Seniors Advisory Board was overall very positive. The Board was rapidly established via the large network of the COOP-consortium. The extensive previous experience of the chairman and senior researchers with PPI, together with professional training in PPI for the junior researcher involved, facilitated fruitful collaboration throughout the project. Digital skills of the Board facilitated convenient online meetings and any expenses could be reimbursed (e.g. for in-person meetings).  Severely frail older people were unfortunately not directly included in the PPI. The Board and the researchers may have missed or misinterpreted their perspectives. Additionally, as the Board also participated in the larger COOP-consortium, their commitment was relatively time-consuming. |
| **Notes**: ^*^According to the Dutch Verhage Scale on educational attainment. **Abbreviations**: COOP, COVID-19 Outcomes in Older People consortium; GRIPP2, Guidance for Reporting Involvement of Patients and the Public version 2; IQR, interquartile range. | |

| **Appendix 3.** Prioritization of the most and least important goals of care, overall and stratified by frailty status. | | | | | |
| --- | --- | --- | --- | --- | --- |
|  | **Overall**  N = 1278 | **Fit**  N = 725 | **Mildly frail**  N = 404 | **Severely frail**  N = 149 | **p-value** |
| **Single most important**, n (%) |  |  |  |  |  |
| Preserving quality of life | 593 (51) | 375 (56) | 167 (47) | 51 (41) | 0.031 |
| Staying independent | 296 (26) | 159 (24) | 106 (30) | 31 (25) |  |
| Preventing nursing home admission | 137 (12) | 80 (12) | 45 (13) | 12 (10) |  |
| Relieving symptoms | 45 (3.9) | 19 (2.8) | 13 (3.6) | 13 (11) |  |
| Preventing hospital admission | 31 (2.7) | 15 (2.2) | 8 (2.2) | 8 (6.5) |  |
| Supporting others | 31 (2.7) | 12 (1.8) | 13 (3.6) | 6 (4.8) |  |
| Extending life | 21 (1.8) | 12 (1.8) | 6 (1.7) | 3 (2.4) |  |
|  |  |  |  |  |  |
| **Single least important**, n (%) |  |  |  |  |  |
| Extending life | 441 (39) | 257 (39) | 135 (39) | 49 (41) | 0.611 |
| Supporting others | 238 (21) | 146 (22) | 71 (20) | 21 (18) |  |
| Preventing hospital admission | 166 (15) | 102 (16) | 49 (14) | 15 (13) |  |
| Preventing nursing home admission | 143 (13) | 78 (12) | 48 (14) | 17 (14) |  |
| Relieving symptoms | 72 (6.4) | 41 (6.3) | 28 (8.0) | 3 (2.5) |  |
| Staying independent | 44 (3.9) | 18 (2.8) | 14 (4.0) | 12 (10) |  |
| Preserving quality of life | 19 (1.7) | 11 (1.7) | 6 (1.7) | 2 (1.7) |  |
|  |  |  |  |  |  |
| **Notes**: Missing data (total n and % of the fit, mildly frail, and severely frail subgroups): most important (103; 6.2%, 8.9% and 15%) and least important (148; 9.2%, 13% and 20%). Otherwise excluded from this analysis (i.e. regarded the self-phrased goal; total n and % of the fit, mildly frail, and severely frail subgroups): most important (21: 1.1%, 2.5% and 2.0%) and least important (7: 0.7%, 0.2% and 0.7%). | | | | | |

| **Appendix 4.** Goals of care stratified by mental and social health problems. | | | | | | |
| --- | --- | --- | --- | --- | --- | --- |
|  | **Mental health problems** | | **p-value** | **Social health problems** | | **p-value** |
|  | **No** | **Yes** |  | **No** | **Yes** |  |
|  | N = 776 | N = 497 |  | N = 874 | N = 402 |  |
|  |  |  |  |  |  |  |
| **Preventing nursing home admission**, n (%) |  |  |  |  |  |  |
| Unimportant | 43 (5.6) | 45 (9.4) | 0.036 | 58 (6.7) | 30 (7.7) | 0.481 |
| Somewhat important | 51 (6.7) | 27 (5.6) |  | 58 (6.7) | 20 (5.2) |  |
| Very important | 672 (88) | 408 (85) |  | 744 (87) | 338 (87) |  |
|  |  |  |  |  |  |  |
| **Staying independent**, n (%) |  |  |  |  |  |  |
| Unimportant | 23 (3.0) | 30 (6.1) | 0.008 | 25 (2.9) | 28 (7.0) | <0.001 |
| Somewhat important | 80 (10) | 63 (13) |  | 112 (13) | 32 (8.0) |  |
| Very important | 672 (87) | 400 (81) |  | 735 (84) | 339 (85) |  |
|  |  |  |  |  |  |  |
| **Preserving quality of life**, n (%) |  |  |  |  |  |  |
| Unimportant | 20 (2.6) | 36 (7.3) | <0.001 | 25 (2.9) | 31 (7.8) | <0.001 |
| Somewhat important | 80 (10) | 77 (16) |  | 102 (12) | 57 (14) |  |
| Very important | 674 (87) | 380 (77) |  | 745 (85) | 310 (78) |  |
|  |  |  |  |  |  |  |
| **Relieving symptoms**, n (%) |  |  |  |  |  |  |
| Unimportant | 49 (6.4) | 47 (9.6) | 0.107 | 55 (6.4) | 41 (10) | 0.016 |
| Somewhat important | 162 (21) | 102 (21) |  | 173 (20) | 91 (23) |  |
| Very important | 560 (73) | 342 (70) |  | 637 (74) | 267 (67) |  |
|  |  |  |  |  |  |  |
| **Preventing hospital admission**, n (%) |  |  |  |  |  |  |
| Unimportant | 98 (13) | 74 (15) | 0.322 | 122 (14) | 50 (13) | 0.469 |
| Somewhat important | 127 (17) | 88 (18) |  | 153 (18) | 63 (16) |  |
| Very important | 547 (71) | 329 (67) |  | 591 (68) | 286 (72) |  |
|  |  |  |  |  |  |  |
| **Supporting others**, n (%) |  |  |  |  |  |  |
| Unimportant | 76 (9.8) | 83 (17) | 0.001 | 91 (11) | 68 (17) | <0.001 |
| Somewhat important | 179 (23) | 109 (22) |  | 189 (22) | 99 (25) |  |
| Very important | 517 (67) | 299 (61) |  | 586 (68) | 232 (58) |  |
|  |  |  |  |  |  |  |
| **Extending life**, n (%) |  |  |  |  |  |  |
| Unimportant | 278 (36) | 242 (49) | <0.001 | 316 (36) | 205 (52) | <0.001 |
| Somewhat important | 227 (29) | 123 (25) |  | 260 (30) | 92 (23) |  |
| Very important | 267 (35) | 125 (26) |  | 294 (34) | 98 (25) |  |
|  |  |  |  |  |  |  |
|  |  |  |  |  |  |  |
| **Single most important**, n (%) |  |  |  |  |  |  |
| Preserving quality of life | 396 (56) | 196 (44) | 0.002 | 447 (56) | 146 (41) | <0.001 |
| Staying independent | 174 (25) | 122 (28) |  | 180 (23) | 116 (33) |  |
| Preventing nursing home admission | 74 (10) | 62 (14) |  | 94 (12) | 43 (12) |  |
| Relieving symptoms | 23 (3.2) | 22 (5.0) |  | 29 (3.6) | 16 (4.5) |  |
| Preventing hospital admission | 17 (2.4) | 14 (3.2) |  | 14 (1.8) | 17 (4.8) |  |
| Supporting others | 12 (1.7) | 19 (4.3) |  | 18 (2.3) | 13 (3.6) |  |
| Extending life | 14 (2.0) | 7 (1.6) |  | 15 (1.9) | 6 (1.7) |  |
|  |  |  |  |  |  |  |
| **Single least important**, n (%) |  |  |  |  |  |  |
| Extending life | 264 (39) | 176 (41) | 0.402 | 287 (37) | 153 (44) | 0.114 |
| Supporting others | 156 (23) | 82 (19) |  | 164 (21) | 74 (21) |  |
| Preventing hospital admission | 103 (15) | 61 (14) |  | 129 (17) | 37 (11) |  |
| Preventing nursing home admission | 85 (12) | 58 (13) |  | 98 (13) | 45 (13) |  |
| Relieving symptoms | 46 (6.7) | 26 (6.0) |  | 52 (6.7) | 20 (5.7) |  |
| Staying independent | 21 (3.1) | 23 (5.3) |  | 31 (4.0) | 13 (3.7) |  |
| Preserving quality of life | 11 (1.6) | 8 (1.8) |  | 11 (1.4) | 8 (2.3) |  |
|  |  |  |  |  |  |  |
|  | | | | | | |

**References**

1. ZonMw. COVID19 Outcomes in Older People - the COOP study. https://projecten.zonmw.nl/en/project/covid19-outcomes-older-people-coop-study (5 March 2024, date last accessed)

2. Rijksoverheid. Coronavirus tijdlijn: Ontwikkelingen coronavirus in 2022 [Coronavirus timeline: Developments coronavirus in 2022]. <https://www.rijksoverheid.nl/onderwerpen/coronavirus-tijdlijn/2022> (30 March 2023, date last accessed)

3. Rijksoverheid. COVID-19 vaccinaties [COVID-19 vaccinations]. https://coronadashboard.government.nl/landelijk/vaccinaties (30 March 2023, date last accessed)

4. Nieuwsuur. Corona playlist of the news of the Dutch Public Broadcastor. Video: 'Kijk maar mee als je denkt dat het allemaal wel meevalt' ['Watch this if you think it is not that serious'] <https://www.youtube.com/watch?v=XYmbGG6Qlmw&list=PLxtc6c2-jvSAFJ_SX-lLPi-GomSIQRVj0> (30 March 2023, date last accessed)

5. Thuisarts.nl. Patient information website from the Dutch General Practitioners Association. Ik ben oud en heb een zwakke gezondheid en denk na over behandeling bij corona [I am old and have poor health and am considering treatment for corona]. 17-05-2021. <https://www.thuisarts.nl/ik-heb-corona/ik-ben-oud-en-heb-zwakke-gezondheid-en-denk-na-over-behandeling-bij-corona> (30 March 2023, date last accessed)

6. Zembla. Onafhankelijke onderzoeksjournalistiek: hoe denken ‘kwetsbare’ ouderen zelf over de coronacrisis? [independent research journalism: how do "frail" older adults themselves think about the coronacrisis?]. 20-05-2021. <https://www.bnnvara.nl/zembla/artikelen/hoe-denken-kwetsbare-ouderen-zelf-over-de-coronacrisis> (30 March 2023, date last accessed)

7. Boddaert M, Caffarel J, Dermois M ea. Leidraad voor het proces en uniform vastleggen van proactieve zorgplanning naar aanleiding van de COVID-19-pandemie [Guideline for the process of and unequivocal documentation of advanced care planning in response to the COVID-19 pandemic]. 22-10-2020. <https://palliaweb.nl/getmedia/1e1ce663-6f7d-46ac-a47a-c8cb589597b7/Leidraad-proactieve-zorgplanning-(ACP)_22102020_new.pdf> (30 March 2023, date last accessed)

8. Qualtrics software, version May 2022 of Qualtrics. Copyright © 2020 (Provo, UT, USA). Available at: <https://www.qualtrics.com/>.

9. Moens IS, van Gerven LJ, Debeij SM, et al.; Positive health during the COVID-19 pandemic: a survey among community-dwelling older individuals in the Netherlands. *BMC Geriatr* 2022;**22**(1):51. doi: 10.1186/s12877-021-02737-2.

10. van der Klei VMGTH, Moens IS, Simons T, et al.; The impact of the COVID-19 pandemic on Positive Health among older adults in relation to the complexity of health problems. *Journal of the American Geriatrics Society*;**n/a**(n/a). doi: <https://doi.org/10.1111/jgs.18695>.

11. Church S, Rogers E, Rockwood K, et al.; A scoping review of the Clinical Frailty Scale. *BMC Geriatr* 2020;**20**(1):393. doi: 10.1186/s12877-020-01801-7.

12. Blom J, den Elzen W, van Houwelingen AH, et al.; Effectiveness and cost-effectiveness of a proactive, goal-oriented, integrated care model in general practice for older people. A cluster randomised controlled trial: Integrated Systematic Care for older People--the ISCOPE study. *Age Ageing* 2016;**45**(1):30-41. doi: 10.1093/ageing/afv174.

13. NHS Acute Frailty Network. The Clinical Frailty Scale (CFS): A Quick Reference Guide. https://www.acutefrailtynetwork.org.uk/uploads/files/1/CFS%20Quick%20Reference%20Guide%20FINAL.pdf

14. Theou O, Pérez-Zepeda MU, van der Valk AM, et al.; A classification tree to assist with routine scoring of the Clinical Frailty Scale. *Age Ageing* 2021;**50**(4):1406-1411. doi: 10.1093/ageing/afab006.

15. Rockwood K, Song X, MacKnight C, et al.; A global clinical measure of fitness and frailty in elderly people. *Cmaj* 2005;**173**(5):489-95. doi: 10.1503/cmaj.050051.

16. Etkind SN, Lovell N, Bone AE, et al.; The stability of care preferences following acute illness: a mixed methods prospective cohort study of frail older people. *BMC Geriatr* 2020;**20**(1):370. doi: 10.1186/s12877-020-01725-2.

17. Kaldjian LC, Curtis AE, Shinkunas LA, et al.; Goals of care toward the end of life: a structured literature review. *Am J Hosp Palliat Care* 2008;**25**(6):501-11. doi: 10.1177/1049909108328256.

18. Verhage F. *Intelligentie en leeftijd onderzoek bij Nederlanders van twaalf tot zevenenzeventig jaar [Intelligence and age: Research study in Dutch individuals aged twelve to seventy-seven].* Assen: Van Gorcum., 1964.

19. Staniszewska S, Brett J, Simera I, et al.; GRIPP2 reporting checklists: tools to improve reporting of patient and public involvement in research. *BMJ* 2017;**358**:j3453. doi: 10.1136/bmj.j3453.

20. Smits DW, van Meeteren K, Klem M, et al.; Designing a tool to support patient and public involvement in research projects: the Involvement Matrix. *Res Involv Engagem* 2020;**6**:30. doi: 10.1186/s40900-020-00188-4.
